# Implementation and Evaluation of a Cancer Immunotherapy Elective for Medical Students: Mixed Methods Descriptive Study

**DOI:** 10.2196/71628

**Published:** 2026-01-21

**Authors:** Mark Raynor, Rivers Hock, Brandon Godinich, Satish Maharaj, Houriya Ayoubieh, Cynthia Perry, Jessica Chacon

**Affiliations:** 1 Department of Medical Education, Foster School of Medicine, Texas Tech University Health Sciences Center El Paso, 130 Rick Francis, El Paso, TX, 79905, United States, 1 915-215-6116; 2 Department of Hematology/Oncology, University of South Florida, Tampa, FL, United States; 3 Moffitt Cancer Center, Tampa, FL, United States

**Keywords:** medical education, immunotherapy, cancer, undergraduate medical education, cancer immunotherapy

## Abstract

**Background:**

Cancer immunotherapy represents a transformative advancement in oncology, offering new avenues for treating malignancies by harnessing the immune system. Despite its growing clinical relevance, immunotherapy remains underrepresented in undergraduate medical education, particularly in curricula integrating foundational immunology with clinical application. To address this gap, we developed and implemented a fully online elective for fourth-year medical students focused on core immunology concepts, immunotherapy mechanisms, Food and Drug Administration–approved treatments, immune-related adverse events, and patient-centered therapeutic decision-making.

**Objective:**

This study aimed to evaluate the effectiveness of an asynchronous-synchronous online cancer immunotherapy elective in improving medical student knowledge, engagement, and critical-thinking skills. We hypothesized that participation in the elective would be associated with perceived improvements in knowledge and clinical preparedness and inform future strategies for integrating cancer immunotherapy into medical curricula.

**Methods:**

We conducted a mixed methods study with fourth-year medical students enrolled in a 2-week elective at a US medical school. The curriculum included a self-paced foundational module, online discussion board, and a capstone oral presentation requiring students to propose a novel immunotherapy approach. Participants completed pre- and postcourse quizzes assessing immunotherapy knowledge and an anonymous postcourse Likert-scale survey. Quantitative data were summarized descriptively, and Likert responses were reported using medians and IQRs. Due to the small sample size, 2-tailed unpaired *t* tests comparing pre- and postcourse quiz averages were underpowered to detect statistically significant differences. Qualitative data were analyzed using inductive thematic analysis with investigator triangulation.

**Results:**

A total of 35 students completed the elective, and 20 submitted the postcourse survey (response rate: 57%). Across all Likert-scale items, students reported a median response of 5 (Strongly Agree), with IQR values ranging from 0 to 1, indicating uniformly positive perceptions and minimal variability in their evaluation of the course. Descriptively, average postcourse quiz scores were higher than precourse scores, suggesting improved conceptual understanding. Qualitative thematic analysis revealed three major themes: (1) increased confidence engaging with complex immunotherapy mechanisms, (2) appreciation for the flexibility and interactivity afforded by the hybrid asynchronous-synchronous model, and (3) enhanced understanding of the real-world clinical application of immunotherapy across interdisciplinary settings.

**Conclusions:**

Descriptive quantitative and qualitative findings suggest that a targeted online cancer immunotherapy elective may enhance learners’ perceived knowledge and critical-thinking capacity related to emerging cancer therapies. The course’s hybrid structure offered flexibility, accessibility, and potential scalability. As immunotherapy continues to expand in clinical practice, this model provides a promising framework for integration into medical curricula. Future work should include larger cohorts and longitudinal follow-up into residency to more rigorously assess educational impact.

## Introduction

Immunotherapy, heralded as a beacon of hope in the oncological landscape, stands at the forefront of medical innovation, offering novel avenues for combating cancer [1-3]. As immune checkpoint blockade, cellular therapies, and cancer vaccinology assume increasing prominence, proficiency in cancer immunotherapy is becoming an essential competency for future health care practitioners [1,2,4,5]. Yet, despite its transformative clinical impact, formal education on cancer immunotherapy remains fragmented or absent in most undergraduate medical curricula. Prior studies have highlighted that health care trainees often feel underprepared to discuss or apply immunotherapy principles in clinical settings, reflecting a broader disconnect between rapid scientific advances and medical education [6,7].

Existing educational work in this area has largely centered on postgraduate oncology programs, continuing medical education, or highly specialized workshops. There remains a critical gap in evidence-based, integrative, and educational models that can effectively prepare medical students, before residency, for the clinical realities of modern cancer care. This lack of structured exposure creates downstream challenges: learners enter rotations or residency without foundational immunotherapy literacy, clinicians report variability in trainee preparedness, and the rapidly evolving therapeutic landscape continues to outpace traditional curricula.

To address this gap, we developed the *Basics and Implementation of Cancer Immunotherapy* elective at the Texas Tech University Health Sciences Center (TTUHSC) El Paso Paul L. Foster School of Medicine. This fully online elective was intentionally designed to provide accessible foundational knowledge while fostering higher-order thinking, clinical reasoning, and innovation in the context of rapidly evolving immunotherapy treatments.

The goal of the course was to deliver content and create a sustainable, adaptable, and evaluable model for broader implementation. Therefore, we grounded the curriculum in 2 complementary frameworks: RE-AIM (Reach, Effectiveness, Adoption, Implementation, and Maintenance) and Kirkpatrick’s model. RE-AIM is an implementation science framework typically used in public health and program dissemination research [8,9]. It guided the structural design of the elective by prompting us to consider who the elective could reach, how well it might function across learner groups, what resources and faculty expertise were required for adoption, how consistently it could be delivered in an online environment, and how it could be maintained or scaled over time.

In contrast, Kirkpatrick’s model, which focuses on Reaction, Learning, Behavior, and Results, provided a pedagogical framework for evaluating educational impact at the learner level. This dual framework approach allowed us to simultaneously examine (1) learner satisfaction, knowledge, and perceived skill development, and (2) broader programmatic considerations such as feasibility, reproducibility, and sustainability within an already crowded medical curriculum [10-14]. By integrating these 2 models, we aimed to ensure both a high-quality educational experience and a design that could be reliably implemented or adapted by other institutions.

This study aimed to evaluate the effectiveness of the elective in enhancing student knowledge and critical thinking using a mixed methods approach. Specifically, we sought to answer the research question: Does participation in this elective improve students’ knowledge, perceptions of course relevance, and critical thinking in cancer immunotherapy? We hypothesized that students completing the elective would demonstrate measurable knowledge gains, report a positive educational experience, and show enhanced critical thinking related to immunotherapy. Findings from this study can guide future curricular efforts aimed at integrating contemporary immunotherapy concepts into undergraduate medical education in a structured and measurable way.

## Methods

### Rationale for Elective Development

Medical students at Foster School of Medicine receive limited exposure to cancer immunotherapy during the preclerkship phase because the integrated curriculum and density of required content constrain opportunities for in-depth coverage [15-17]. To address this gap, faculty designed a fourth-year elective grounded in a spiral learning model that builds on students’ foundational immunology knowledge from the first 2 preclerkship years and reinforces concepts introduced during their clinical rotations.

### Curricular Design

We selected a virtual elective to match student preferences for greater fourth-year flexibility and to leverage the institution’s expanded capacity for high-quality online learning primarily developed during the COVID-19 era [18-21]. The elective directly addressed institutional needs identified through annual curriculum review and supported the School of Medicine’s priority to prepare graduates for rapidly evolving biomedical innovations, such as immunotherapy. Within the Department of Medical Education, the elective advanced key curricular priorities: (1) integrating foundational basic sciences, such as immunology, with clinical reasoning; (2) broadening elective options that promote scholarly inquiry; and (3) expanding technology-enhanced and competency-based instruction.

Faculty with expertise in cancer immunotherapy, biochemistry and cell biology, clinical genetics, oncology, educational design, and online pedagogy led curriculum development. The team followed a staged design process: we mapped core immunotherapy competencies, identified gaps across the preclinical and clerkship curriculum, and iterated content through consultation with our clinical oncology faculty. Medical students actively contributed to early course development. A small group of students co-developed the immunology primer by drawing on their research experience and peer and faculty feedback to ensure clarity, relevance, and appropriate depth. These students also conducted an informal needs assessment through conversations with classmates, confirming that many trainees felt underprepared to discuss contemporary cancer immunotherapies during clinical rotations.

The faculty built the final course using interactive synchronous and asynchronous components, including the student-developed primer, structured discussion boards, and a culminating student presentation. These elements aimed to deepen engagement with the scientific literature and support inquiry–driven learning [18-20]. Constructivist learning theory, interdisciplinary integration [22], and reflective practice guided the instructional approach. Expert consensus, national immunotherapy competency frameworks, and student-identified needs informed topic selection. Basic science and clinical faculty peer-reviewed all content before launch to ensure scientific rigor, clinical relevance, and instructional quality.

### Pedagogical Framework, Principles, and Competencies and Standards

Developing a fourth-year elective for medical students on cancer immunotherapy required deliberate alignment with pedagogical frameworks, instructional principles, established competencies, and educational standards to meet the learning objectives. Basic science faculty (immunology, biochemistry, and cell biology) and clinical faculty (internal medicine, oncology, and clinical genetics) jointly oversaw the course.

### Constructivist and Experiential Foundations

The design of the elective drew on constructivist and experiential learning theories to promote knowledge construction through active engagement, inquiry, and reflective practice. We incorporated Kirkpatrick’s Four-Level Model and the RE-AIM framework to guide course design and evaluation. These frameworks ensured that learning activities targeted measurable outcomes, real-world application, and iterative improvement based on faculty and student feedback. The course combined asynchronous modules, faculty-led synchronous discussions, and a final oral presentation to support both individual learning and collaborative inquiry.

#### Kirkpatrick’s Four-Level Model

We applied the Kirkpatrick’s Model [10] to evaluate student learning and course impact.

- Level 1. Reaction: We collected postcourse survey feedback to assess engagement and satisfaction with course structure and content.
- Level 2. Learning: We measured knowledge acquisition using asynchronous pre- and postquizzes focused on immunology basics and the interplay between the immune system and the tumor microenvironment.
- Level 3. Behavior: We evaluated critical thinking by assigning discussion prompts requiring students to propose novel therapies and apply theoretical concepts in innovative ways.
- Level 4. Results: We examined how effectively students identified and proposed solutions to gaps in current immunotherapy practices during their oral presentations.

#### RE-AIM Framework

In parallel, we used the RE-AIM framework to evaluate implementation and scalability.

- *Reach*: We monitored enrollment numbers and demographic diversity.
- *Effectiveness*: We analyzed improvements in quiz scores and qualitative feedback on learning outcomes.
- *Adoption*: We noted the course’s integration into the fourth-year elective catalog and student uptake across multiple intended specialties.
- *Implementation*: We assessed delivery fidelity, faculty coordination, and alignment of synchronous and asynchronous components.
- *Maintenance*: We planned annual updates to incorporate emerging immunotherapy advances and maintain curricular relevance.

This dual framework approach enabled a comprehensive evaluation of learner experience and overall educational impact.

##### Pedagogical Framework

We provided a pedagogical framework for evaluating educational impact at the learner level:

- *Constructivist approach* [23]: Active learning was emphasized where students constructed their understanding of cancer immunotherapy through exploration, inquiry, and problem-solving.
- *Interdisciplinary integration* [24]: Principles from basic sciences, such as immunology, cancer biology, pharmacology, and molecular biology, were integrated to provide a holistic understanding of cancer immunotherapy.
- *Reflective practice* [25,26]: Students were encouraged to reflect on their learning, clinical experiences, and possible immune adverse events. Students were additionally challenged to consider their patient population, particularly those underrepresented in medicine when considering immunotherapy for cancer treatment.

##### Principles

Principles were integrated as follows:

- *Evidence-based* [27]: All content and teaching methods were grounded in current scientific evidence and clinical practice guidelines. A consultation with experts in the cancer immunotherapy field at the basic science and clinical settings was conducted.
- *Patient-centered* [28,29]: The importance of patient perspectives and personalized treatment approaches in cancer care was emphasized.
- *Ethical awareness* [30]: Ethical dilemmas, such as access to treatment, clinical trial participation, and the implications and importance of genetic testing in cancer immunotherapy, were discussed.

##### Competencies and Standards [31-34]

We developed the competencies and standards to meet the learning objectives:

- *Knowledge base*: Ensure that students understand the fundamental principles of cancer immunology, including the role of immune checkpoints, innate and adaptive immunity, cytokines, and monoclonal antibodies in cancer treatment.
- *Clinical skills*: Ensure that students develop a foundational understanding of how clinicians evaluate candidates for immunotherapy, monitor treatment responses, and identify common immune-related adverse events.
- *Critical thinking*: Oversee student ability to analyze current immunotherapy options and formulate evidence-based innovative treatment.
- *Communication skills*: Provide feedback on student ability to effectively communicate with faculty and peers regarding basic science concepts, cancer immunotherapy treatment, and potential side effects.
- *Professionalism*: Provide student feedback on demonstrating empathy, cultural competence, and ethical behavior in all aspects of immunotherapy care and professional interactions.

##### Elective Structure

We developed the elective structure for enhancing student knowledge and critical thinking:

- *Pre-elective quiz*: We developed the pre-elective quiz to measure students’ baseline understanding of basic immunology and foundational cancer immunotherapy concepts. Faculty selected quiz topics by mapping them to the elective’s core learning objectives and to national immunotherapy competency frameworks [35,36]. The prequiz intentionally emphasized basic foundational knowledge, including innate and adaptive immunity, immune cell function, antigen recognition, tumor and immune system interactions, and introductory immunotherapy mechanisms, to ensure that all learners entered the elective with a minimum level of readiness. To determine the length of the quiz, faculty reviewed comparable formative and summative assessments used in preclerkship immunology courses at TTUHSC El Paso and agreed on a concise 15-item structure that would prevent test fatigue while allowing coverage of core concepts. Before administering the quiz, basic science faculty and clinical faculty members conducted a peer review to evaluate question clarity, clinical accuracy, and alignment with learning outcomes. The team performed an informal bias and sensitivity review to ensure that items did not disadvantage students based on specialty interest or prior research experience. Faculty also performed a preliminary item difficulty appraisal using standard immunology benchmarks to ensure that the quiz appropriately captured foundational, not advanced, knowledge. Students completed the pre-elective quiz electronically on the first day of the course. Faculty used group-level response patterns to identify shared conceptual gaps and to tailor emphasis during synchronous discussions.

- *Immunology primer review*: Students reviewed a self-paced immunology primer that reinforced foundational concepts needed to analyze immunotherapy mechanisms and clinical applications. The primer served as the common baseline for all subsequent discussions.

- *Introduction to cancer immunotherapy assigned readings*: Assigned readings introduced the historical evolution of immunotherapy, early discoveries in tumor immunology, and the scientific rationale behind modern immune-based cancer treatments. Students were expected to extract key mechanistic principles and discuss them during faculty-facilitated sessions.

- *Interactive discussion board (collaborative inquiry)*: Over 2 weeks, students participated in a moderated online discussion board (Canvas LMS). Faculty posted weekly prompts connecting mechanistic immunology to clinical controversies (eg, “Should combination immunotherapy be used upfront in all patients with high tumor burden?”). Students responded to peers, synthesized evidence from the literature, and reflected on the ethical and clinical implications of their arguments. Faculty monitored discussions, asked probing questions, and clarified misconceptions in real time.

Faculty facilitators led structured online discussions using a consistent set of prompts, such as the following:

- What immunologic mechanism explains this patient’s response or nonresponse?
- What biomarkers would guide treatment selection?
- How would you monitor for immune-related adverse events, and which patient population is at most risk?
- What therapeutic alternatives exist, and what is the evidence supporting them?

This structure ensured clinically rigorous, evidence-driven dialogue while encouraging students to integrate mechanistic immunology with real-world oncology decision-making.

- *Ethical considerations, patient perspectives, and social determinants of health*: To ensure robust and meaningful discussion of ethics and equity, the faculty developed structured ethical prompts and probes related to access, affordability, and geographic disparities in immunotherapy delivery. Examples included the following: (1) How do insurance status and socioeconomic factors influence a patient’s ability to access checkpoint inhibitors? (2) What ethical considerations arise when offering chimeric antigen receptor (CAR) T-cell therapy to patients in rural or underserved regions? (3) How should clinicians navigate treatment decisions when cultural beliefs or health literacy affects understanding of immunotherapy risks?

- *Future directions and research (student-led inquiry)*: Students explored emerging topics such as next-generation CAR T-cell constructs, tumor microbiome effects on immunotherapy, predictive biomarkers, and novel combination strategies. Faculty provided a curated list of high-impact reviews and seminal papers. Each student selected a topic, analyzed current evidence, evaluated limitations of existing studies, and proposed future research directions and a novel therapy. Students presented their findings during a final oral presentation and received faculty feedback grounded in scientific rigor and clinical relevance.
- *Postelective quiz*: We designed the postelective quiz to parallel the prequiz in structure, length, and difficulty so that performance changes reflected student learning rather than differences in test format. Like the prequiz, the postquiz included 15 multiple-choice questions aligned with the same foundational and applied immunotherapy concepts. To ensure content validity, basic science and clinical faculty mapped each postquiz item to the learning objectives addressed during the elective, including immune mechanisms of action, indications for Food and Drug Administration (FDA)–approved immunotherapies, and immune-related adverse events. Faculty conducted the same peer review, clarity check, and bias review procedures used for the prequiz. Student anonymity prevented individual-level tracking; therefore, we analyzed quiz results at the aggregate group level, comparing pre- and postcourse means to identify trends in knowledge acquisition. Students completed the postelective quiz immediately after the final oral presentations. Faculty used the results to evaluate learning outcomes, assess overall instructional effectiveness, and identify content areas requiring refinement in future course iterations.

### Primer Development and Foundational Preparation

To strengthen students’ readiness for advanced immunotherapy concepts, we provided a self-paced immunology primer at the start of the course. A basic scientist with immunology expertise and a clinical oncologist collaboratively selected the primer topics to ensure alignment with learning objectives and relevance to both foundational and clinical content. Medical students then drafted and refined the primer content, drawing on their experience in the preclerkship curriculum to identify which concepts were most challenging and most critical for clinical application during the clerkship curriculum. This student-driven development process (1) incorporated learners’ perspectives to better target foundational gaps and (2) supported student contributors’ professional growth through scholarly authorship and early medical education experience. Faculty reviewed and approved all content to ensure accuracy, clarity, and educational value. Integrating student voices added a peer-informed dimension to the primer, helping bridge the gap between didactic instruction and practical understanding.

### Knowledge Assessment

We created pre- and postelective quizzes (Multimedia Appendix 1) to gauge students’ baseline knowledge, measure learning gains, and evaluate the effectiveness of course content and teaching methods. On the first day of the elective, students completed the pre-elective quiz. Afterward, they received the immunology primer (Multimedia Appendix 2) as a foundational resource. The primer introduced key immunology topics, including innate and adaptive immunity, immune-cell functions, and mechanisms of immune activation. It emphasized how these principles govern the detection and elimination of cancer cells and highlighted the interplay between the immune system and the tumor microenvironment. The primer also incorporated recent advances such as checkpoint inhibitors and CAR T-cell therapy, enabling students to connect core immunological principles to cutting-edge cancer treatments. By reinforcing these concepts, the primer equipped students with the background needed to understand the complexities of cancer immunotherapy.

Table 1 outlines the key topics and learning objectives that guided students through essential features of the tumor microenvironment and the application of immunotherapy in cancer treatment. Students engaged with these objectives through an online discussion board (Canvas LMS) and delivered an oral presentation on the final day of the elective.

**Table 1.** List of topics and specific objectives for the online discussion and final presentation.

Topic	Objectives
Discuss tumor microenvironment and the basis for using immunotherapy.	Outline the mechanism of how cancer cells lead to T-cell exhaustion. Know the basics of other tumor microenvironment effects such as angiogenesis and extracellular matrix changes.
Describe the mechanism of action of immunotherapy techniques.	Identify the role of PD-1^a^, PD-L1^b^, CTLA-4^c^ inhibitors, and CAR^d^ T-cells in cancer therapy.
What are the most common combinations of immunotherapy drugs used at the present time?	Discuss current effective treatments available such as for melanoma and lung cancer. Identify promising clinical trials and new developments in immunotherapy.
What are the adverse events commonly associated with immunotherapy?	Identify common immunotherapy-related adverse events. Know the current terminology used to classify immunotherapy adverse events.
What patient population is more at risk for adverse events while taking immunotherapy?	Identify risk factors based on patient demographics, underlying medical conditions, and cancer-type specific reactions. Determine whether a patient is a good candidate for immunotherapy given risk factors.
Novel therapies: Research current treatment modalities as well as developments in the field. Propose a new therapy protocol, given your new knowledge of immunotherapy.	Propose a new therapy based on the understanding of immunotherapy mechanisms based on literature review. Differentiate your innovative treatment from what is currently being used or investigated.

a PD-1: programmed death-1.

b PD-L1: programmed death ligand-1.

c CTLA-4: cytotoxic T-lymphocyte antigen 4.

d CAR: chimeric antigen receptor.

### Participants

Participants in this study consisted of fourth-year medical students enrolled at TTUHSC El Paso. We used purposive sampling to target students in their final year who were eligible and available to participate in a 2-week online elective. Students qualified for inclusion in the research study if they voluntarily enrolled in the elective and provided informed consent to participate in the research study by completing postelective surveys. Participation required students to agree to the study procedures and complete all associated assessments. A total of 35 students enrolled in the elective, and 20 of those students completed the postcourse survey.

We focused on fourth-year students because they typically select electives that align with their intended specialty or postgraduate training plans (Table 2). We chose an online format for this elective based on prior institutional success with virtual coursework and to provide maximal flexibility for students who may travel for residency interviews during this period [18].

Table 2 shows the residency programs that students who completed the cancer immunotherapy elective applied to. The distribution illustrates their specialty preferences, with a notably higher number pursuing internal medicine compared with other fields.

**Table 2.** Residency programs medical students completing the cancer immunotherapy elective applied to.

Residency program	Students, n
Internal medicine	5
Pediatrics	3
Anesthesiology	3
Emergency medicine	2
Family medicine	2
Med/Peds	2
Otolaryngology	1
Pathology	1
Psychiatry	1
Total number of students	20

### Assessment Instruments

We assessed students’ knowledge using pre- and postcourse quizzes that immunology and oncology faculty collaboratively designed. Faculty with expertise in immunotherapy mechanisms, curriculum design, and clinical applications created quiz items and aligned them with the elective’s core learning objectives. They also drew on national competency frameworks and relevant literature to support construct and content validity. We evaluated student discussion responses and oral presentations for critical thinking, integration of knowledge, and innovation, providing a complementary behavioral measure aligned with Kirkpatrick level 3 outcomes.

### Survey Instruments

#### Oral Presentation Rubric Design and Validation

We evaluated student presentations using a structured rubric intentionally designed to measure four core constructs aligned with the elective’s learning objectives:

1. *Conceptual understanding*: Accuracy and depth of immunology and immunotherapy knowledge, including mechanisms of action and tumor-immune interactions.
2. *Critical thinking and innovation*: Ability to synthesize literature, evaluate evidence, identify therapeutic gaps, and propose novel or evidence-based strategies.
3. *Clinical application*: Integration of clinical relevance, patient considerations, and implications for treatment decision-making.
4. *Communication and professionalism*: Clarity of oral communication, logical organization, visual design, and professional engagement with peers and faculty.

#### Rubric Development

A team of basic science and clinical faculty collaboratively drafted the rubric. To ensure content validity, faculty mapped each rubric domain directly to the course’s stated competencies and to national guidelines for cancer immunotherapy education. The team reviewed representative presentation topics from prior years to ensure that the rubric captured the range of cognitive skills expected of fourth-year medical students.

#### Validation and Quality Assurance

We strengthened the rubric through a multistep validation process:

- *Peer review*: Three faculty with expertise in immunology, internal medicine, and oncology independently reviewed the rubric for accuracy, alignment with competencies, and clarity of scoring criteria.
- *Bias review*: Educational specialists examined the rubric for ambiguous language, construct-irrelevant variance, and potential bias related to topic selection or communication style.
- *Pilot testing*: Faculty applied the rubric to sample presentation summaries from a previous elective offering. This process allowed the team to refine wording, add performance anchors, and calibrate expectations.
- *Calibration session*: Before evaluation began, faculty reviewers participated in a group-norming session. They scored 2 anonymized sample presentations, compared ratings, and discussed scoring discrepancies to promote interrater consistency.

#### Scoring and Reliability

Two faculty evaluators independently scored each oral presentation using the finalized rubric. We measured interrater reliability by comparing paired scores across all rubric domains. Reviewers resolved discrepancies through discussion until reaching consensus. This dual-review system strengthened score reliability and helped ensure that the rubric consistently captured higher-order reasoning, conceptual accuracy, and clinical integration.

#### Postcourse Survey Instrument

We developed a postcourse survey to evaluate students’ perceptions of the elective and capture qualitative feedback to inform iterative course improvements. We did not use a previously published instrument because no validated tool exists that specifically measures medical students’ perceptions of a cancer immunotherapy elective or assesses learning experiences aligned with RE-AIM and Kirkpatrick evaluation frameworks for a cancer immunotherapy course. Instead, we designed a context-specific instrument grounded in established principles of educational assessment and program evaluation.

The postcourse survey consisted of Likert-scale items, open-ended questions, and demographic prompts and was administered anonymously through Qualtrics. To ensure content validity, the course faculty collaboratively generated the survey items. We mapped each item to the course objectives, the targeted competencies, and the relevant Kirkpatrick levels (Reaction and Learning) to confirm alignment. We refined the survey through faculty peer review, focusing on clarity, item relevance, cognitive load, and potential bias in item phrasing.

To enhance face validity, the draft survey was reviewed by 2 educational researchers unaffiliated with the course who provided feedback on structure, scale selection, and representativeness of domains (usefulness, relevance, comprehension, engagement, and areas for improvement). We revised the instrument accordingly. Due to the small sample size and anonymity requirements, we did not calculate reliability coefficients; however, reviewers assessed the consistency of underlying constructs and the appropriateness of response options.

The final instrument assessed the following domains:

- perception of elective usefulness and relevance;
- comprehension of immunotherapy concepts and techniques;
- engagement with course materials and faculty-led discussions;
- identification of areas requiring improvement; and
- perceived closing of knowledge gaps.

This approach allowed us to capture meaningful student feedback while ensuring that the instrument was appropriate for the elective’s goals and context.

### Data Collection

We administered pre- and postcourse quizzes electronically to assess group-level knowledge acquisition related to basic immunology and cancer immunotherapy. To maintain full anonymity in accordance with institutional review board (IRB) requirements, we collected all quiz responses without any identifiers or linkage markers. No individual-level identifiers were used; therefore, we analyzed all pre- and postcourse quiz data strictly in aggregate and did not attempt to match responses across time points. This approach allowed us to compare group performance before and after the elective while fully protecting student privacy.

Following course completion, we distributed an optional, anonymous postcourse survey through the Qualtrics platform. The survey included Likert-scale items and open-ended prompts assessing students’ perceptions of course effectiveness, relevance of the content, confidence in applying immunotherapy concepts, and suggestions for improvement. Course faculty, including educational researchers, developed the survey to ensure alignment with course objectives and with Kirkpatrick level 1 (Reaction) and level 2 (Learning) outcomes.

### Ethical Considerations

This study was reviewed by the TTUHSC El Paso IRB and determined to meet the criteria for exemption from full board review as a minimal-risk study (protocol no. E23116). In accordance with the approved IRB protocol, the requirement for written informed consent was waived. Participants were provided with an information statement describing the study purpose, planned use of data, and the voluntary nature of participation prior to study involvement. No identifiable information was collected, no compensation was provided, and participant privacy and confidentiality were maintained throughout data collection, analysis, and reporting.

### Quantitative Data Analysis

We used a mixed methods approach to evaluate course outcomes. For the quantitative analysis, we compared group-level pre- and postcourse quiz scores using a 2-tailed unpaired *t* test. We did not link responses at the individual level, so we analyzed all scores in aggregate. The unpaired *t* test allowed us to assess differences in group means while preserving student anonymity.

When designing the knowledge assessment, we did not generate formal power calculations because this elective enrolled a small cohort (n=35), and the study design prioritized anonymity over paired testing, which limited our ability to estimate individual-level effect sizes. Instead, faculty experts in medical education, immunology, internal medicine, and oncology developed the quiz with the expectation, based on content coverage and alignment with the learning objectives, that students would show modest but measurable improvement in foundational immunotherapy knowledge. Given the cohort size and the use of unpaired aggregate scores, the study was underpowered to detect small differences between pre- and postcourse means.

### Qualitative Data Analysis

For qualitative analysis, we examined open-ended survey responses using inductive thematic analysis, following Braun and Clarke’s 6-step methodology [37]. We used Braun and Clarke’s 6-step approach [37] to thematic analysis, a widely applied framework for identifying and interpreting patterns in qualitative data. This process involves becoming familiar with the data, generating initial codes, organizing codes into preliminary themes, refining and reviewing those themes, defining and naming them, and producing the final analytic narrative. We did not apply any a priori theoretical framework; instead, we allowed themes to emerge directly from the data. Two investigators (JC and HA) independently reviewed all responses and generated initial, descriptive codes that reflected recurring ideas in student comments (eg, “clarity of content,” “workload concerns,” “value of clinical discussions,” “ethical considerations,” “confidence applying immunotherapy concepts,” and “suggestions for improvement”). The investigators met to compare and refine these codes, resolve discrepancies through consensus, and organize related codes into preliminary categories (eg, “perceived learning gains,” “course structure,” “engagement and interaction,” and “areas needing clarification”). We iteratively refined a codebook as additional patterns emerged. Thematic saturation occurred when no new codes or concepts appeared in subsequent rounds of review, ensuring that the final themes accurately captured the breadth of student feedback and strengthened the trustworthiness of the analysis.

### Implementing Student Feedback

As part of the postcourse survey, we asked students to identify areas for improvement and propose refinements to the elective. We systematically reviewed all feedback and used it to guide iterative course modifications. Student suggestions directly informed several enhancements implemented in subsequent offerings. For example, many students requested clearer guidance before the oral presentation. In response, we added a detailed grading rubric, sample presentation topics, and a faculty-led question and answer session to clarify expectations. Students also recommended streamlining the weekly workload. To address this, we adjusted reading assignments and aligned asynchronous modules more intentionally with live discussion topics to reduce redundancy and cognitive load.

These changes demonstrated our commitment to continuous quality improvement and student-centered course design. The iterative refinement process, informed by both qualitative and quantitative feedback, reflects the practical application of the RE-AIM and Kirkpatrick frameworks to enhance course delivery and educational effectiveness.

### Study Duration

The study timeline encompassed data collection, analysis, and dissemination of findings over an estimated 1-2 months following the conclusion of the elective. Timely dissemination of findings ensures prompt feedback and informed iterative elective design and delivery improvements, thereby fostering continuous quality enhancement in medical education at TTUHSC El Paso.

## Results

### Knowledge Assessment (Pre- and Postcourse Quiz)

**Figure 1.**
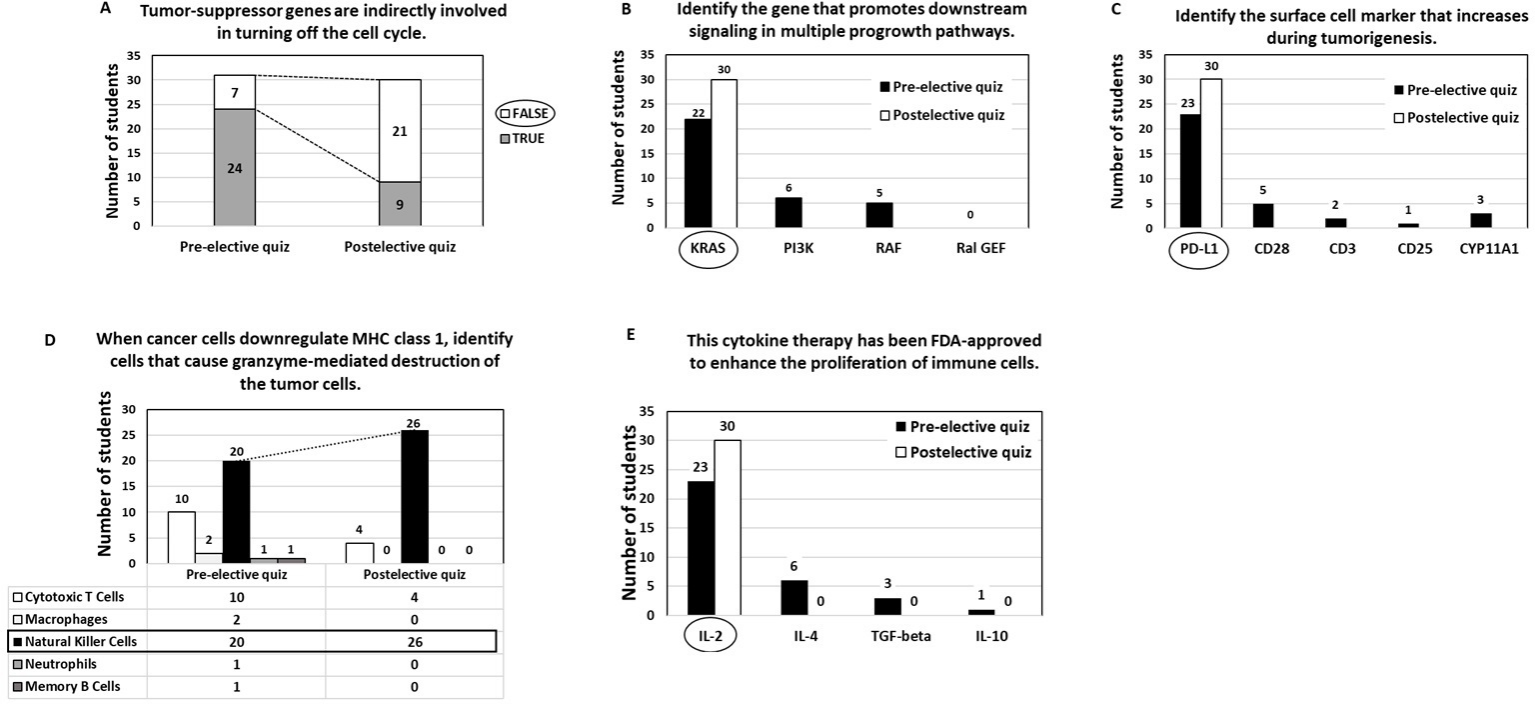
Student proficiency in key concepts of tumor biology and immunotherapy. Compared with pre-elective performance, postelective assessments demonstrated improved student ability to identify and apply foundational concepts in cancer biology and immunotherapy. Specifically, students more accurately recognized the role of tumor-suppressor genes in regulating cell cycle control and preventing malignant transformation (A), as well as the oncogenic function of *KRAS* in driving aberrant intracellular signaling pathways involved in tumor growth and survival (B). In addition, students showed increased understanding of immune regulatory mechanisms within the tumor microenvironment, including the role of PD-L1 in mediating immune checkpoint inhibition (C) and the contribution of natural killer cells to innate antitumor immune responses (D) Students also correctly identified the Food and Drug Administration–approved cytokine therapy used clinically to enhance immune cell proliferation and activity, reflecting improved recognition of immunomodulatory treatment strategies. (E) Together, these results indicate enhanced conceptual understanding across molecular, cellular, and immunologic domains central to contemporary cancer immunotherapy following completion of the elective. FDA: Food and Drug Administration; IL: interleukin-2; MHC: major histocompatibility complex; TGF: transforming growth factor.

A total of 35 students completed the elective, and all quiz data were analyzed in aggregate. Once the students completed the course, they took a postelective quiz. Compared with their performance on the pre-elective quiz, fourth-year medical students demonstrated an improvement in their understanding of basic immunology and cancer immunotherapy topics after completing the elective. For example, students were able to accurately identify the role of tumor-suppressor genes (Figure 1A), the function of *KRAS* (Figure 1B), and the roles of PD-L1 (Figure 1C) and Natural Killer cells (Figure 1D) within the tumor microenvironment. In addition, students could correctly pinpoint which cytokine therapy was FDA-approved for cancer treatment (Figure 1E).

Figure 1 illustrates the enhanced understanding and knowledge gained by students in these critical areas of cancer biology and immunotherapy after completing the elective. Although the 2-tailed unpaired *t* test did not show statistical significance, postelective quiz scores trended higher than pre-elective scores, suggesting a potential improvement in knowledge. This trend supports the educational value of the elective and highlights the need for further study with paired data.

The study design did not link responses at the individual level, and no power calculation was conducted to estimate detectable effect size; therefore, the analysis remained exploratory rather than inferential. The aggregate data nevertheless provided useful information about baseline and end-of-course performance and will guide future refinement of assessment methods, including the use of paired data and sample-size estimation.

### Postcourse Survey

**Figure 2.**
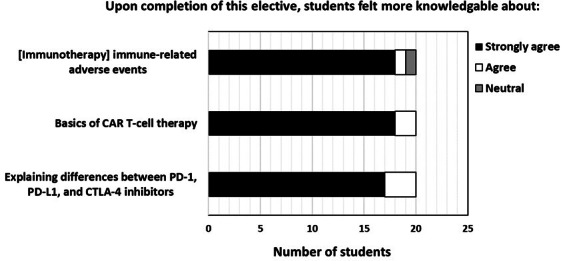
Exploring medical students’ understanding of immune-related adverse events and cancer immunotherapy treatments. Students felt knowledgeable about various types of immune-related adverse events observed in some patients receiving immunotherapy (top). Students felt more knowledgeable about the basics of CAR T-cell therapy (middle). All students agreed that the cancer immunotherapy course enabled them to explain the differences between PD-1, PD-L1, and CTLA-4 (bottom). CAR: chimeric antigen receptor; CTLA-4, cytotoxic T-lymphocyte antigen 4; PD-1: programmed death-1; PD-L1, programmed death ligand-1.

A total of 35 students completed the elective, and 20 students completed the postcourse survey. Figure 2 and Multimedia Appendix 3 summarize the distribution of responses. Students consistently rated the elective favorably across all surveyed domains.

Evaluation of student perceptions demonstrated meaningful gains in their understanding of immune-related adverse events and core immunotherapy mechanisms. Students expressed increased confidence in identifying and describing the types of immune-related adverse events observed in patients undergoing immunotherapy (Figure 2, top). They also reported greater mastery of fundamental CAR T-cell principles (Figure 2, middle). Importantly, every student agreed that the course improved their ability to distinguish between programmed death-1 (PD-1), programmed death ligand-1 (PD-L1), and cytotoxic T-lymphocyte antigen 4 (CTLA-4) (Figure 2 bottom).

Students agreed or strongly agreed that the course was instrumental in learning current immunotherapy techniques (Multimedia Appendix 3A). Students highlighted that the course provided a solid foundation and deeper understanding of cutting-edge immunotherapy practices. Overall, students felt well prepared to apply these techniques in real-world settings.

In addition, the course significantly enhanced students’ ability to think critically about developing novel cancer immunotherapies. Nineteen out of twenty students agreed or strongly agreed that the course’s comprehensive curriculum and emphasis on innovative thinking fostered their analytical skills and creativity (Multimedia Appendix 3B). Through understanding basic science concepts and reviewing the current literature, students were encouraged to explore and devise new strategies for cancer treatment. This critical thinking approach not only deepened their understanding of current immunotherapy techniques but also inspired them to contribute to future advancements in the field.

Finally, in a unanimous consensus, medical students have expressed overwhelming satisfaction with the comprehensive coverage of cancer immunotherapy in their recent course. Each student acknowledged that the course effectively addressed and filled significant gaps in their understanding of immunotherapy (Multimedia Appendix 3C). From foundational concepts to advanced applications, the material provided clarity on complex mechanisms and cutting-edge research in the field. This collective sentiment underscores the course’s success in equipping future health care professionals with essential knowledge and skills crucial for addressing the challenges of modern cancer treatment.

Twenty students completed the postelective survey. Table 3 shows median scores with IQRs for all Likert-scale items. Students reported overwhelmingly positive perceptions of the cancer immunotherapy elective, with consistently high ratings across all survey items. For every item, the median response was 5 (Strongly agree), indicating strong endorsement of the course’s effectiveness. Students agreed that the elective was useful for their learning regarding current immunotherapy techniques (median5, IQR 0) and that the introductory immunology overview supported their understanding of later content (median 5, IQR 0.25).

Students also felt that the course enhanced their ability to think critically about developing novel immunotherapy approaches (median 5, IQR 0) and improved their ability to explain differences among PD-1, PD-L1, and CTLA-4 inhibitors (median 5, IQR 0). Similarly, learners reported increased knowledge of CAR T-cell therapy (median 5, IQR 0) and felt that the amount of course material was appropriate to address gaps in their prior knowledge (median 5, IQR 0).

Students further indicated that their understanding of immune-related adverse events improved as a result of the elective (median 5, IQR 0). The only item with notable response variability was students’ confidence in identifying steps needed to manage patients receiving immunotherapy during residency (median 5, IQR 1.0), suggesting greater heterogeneity in perceived readiness for clinical application.

Overall, these results demonstrate consistently strong satisfaction with the elective and meaningful perceived gains in foundational and applied cancer immunotherapy knowledge. Collectively, these findings suggest high perceived educational value and alignment between course objectives and learner-reported outcomes. A summary of the highest-rated domains is demonstrated in Table 3.

**Table 3.** Twenty students completed the postelective survey^a^.

Item	Median	IQR
Q1. Course usefulness for learning immunotherapy techniques.	5	0
Q2. Immunology overview helped understanding.	5	0.25
Q3. Course enabled critical thinking about novel immunotherapy approaches.	5	0
Q4. Ability to explain PD-1^b^, PD-L1^c^, and CTLA-4^d^ differences.	5	0
Q5. Increased knowledge of CAR^e^ T-cell therapy.	5	0
Q6. Amount of information was appropriate.	5	0
Q7. Increased knowledge of immune-related adverse events.	5	0
Q8. Ability to identify steps to manage patients receiving immunotherapy.	5	1.0

a Median scores and IQRs indicate overwhelmingly positive perceptions of the elective, with all items receiving a median of 5 (Strongly agree). These findings reflect strong alignment between the course objectives and students’ reported gains in cancer immunotherapy knowledge.

b PD-1: programmed death-1.

c PD-L1: programmed death ligand-1.

d CTLA-4: cytotoxic T-lymphocyte antigen 4.

e CAR: chimeric antigen receptor.

### Qualitative Themes From Open-Ended Responses

Inductive thematic analysis of open-ended survey responses revealed 4 dominant themes that collectively illustrate how students experienced the elective:

- Theme 1: Increased Conceptual Clarity reflected students’ reports of gaining a clearer understanding of immune checkpoint pathways, mechanisms of T-cell activation, and distinctions among PD-1, PD-L1, and CTLA-4.
- Theme 2: Strengthened Ability to Apply Concepts Clinically captured students’ increased confidence in connecting mechanistic immunology to therapeutic decision-making, recognition of immune-related adverse events, and understanding FDA-approved therapies such as CAR T-cell therapy.
- Theme 3: Value of Structured, Faculty-Guided Discussion highlighted the importance of guided prompts and faculty facilitation in promoting deeper inquiry and enabling sustained dialogue around ethics, access to care, cost barriers, disparities in clinical trials, and social determinants of health shaping immunotherapy outcomes.
- Theme 4: Recommendations for Course Enhancement included requests for clearer expectations for oral presentations, streamlined weekly readings, and expanded case-based discussions, particularly those incorporating literature focused on marginalized populations.

These qualitative insights not only informed targeted course refinements but also aligned with the evaluative dimensions of the RE-AIM and Kirkpatrick models described in the “Methods” section. Students also expressed strong appreciation for the structured discussion prompts, faculty engagement, and the primer’s effectiveness in scaffolding learning.

## Discussion

### Principal Findings

This mixed methods evaluation demonstrated that a fourth-year online cancer immunotherapy elective improved students’ foundational understanding of immunotherapy principles, strengthened their ability to apply mechanistic knowledge to clinical scenarios, and enhanced their confidence discussing emerging therapies. Students showed measurable gains in core immunology and immunotherapy concepts on the postelective knowledge assessment, and their final oral presentations provided evidence of higher-order reasoning, integration of basic and clinical science, and accurate articulation of complex mechanisms such as PD-1/PD-L1 and CTLA-4 pathways, CAR T-cell engineering, and immune-related toxicities. Qualitative analysis of postelective survey responses further revealed that students valued the elective’s relevance, clarity, and emphasis on clinical translation, noting that it addressed significant gaps in their prior training and supported their ability to think critically about current and emerging cancer immunotherapies. Collectively, these findings indicate that the elective achieved its intended goal of enhancing immunotherapy competency among senior medical students.

### Implications of Findings

The findings from this evaluation demonstrate that a structured, 2-week online cancer immunotherapy elective can enhance medical students’ self-reported understanding of fundamental immunotherapy concepts, mechanisms of action, and FDA-approved treatments. Students also indicated that the elective strengthened their ability to critique scientific literature and articulate clinical applications, skills reinforced through structured discussion prompts and a scored oral presentation. These results suggest that even a short, focused elective can support competency development in emerging therapeutic domains.

The mixed methods data further indicate that pairing asynchronous foundational content (eg, the immunology primer) with guided synchronous discussions and a final presentation provides an effective instructional sequence for fourth-year learners. Students consistently rated the course as useful, relevant, and appropriately rigorous, reflecting the value of integrating preexisting knowledge, analytic tasks, and application-based assessments. This supports the feasibility of using similar instructional designs to introduce other rapidly evolving biomedical topics.

In addition, the elective’s structure, which included evidence-based discussion prompts and opportunities for students to explain mechanisms to peers, offered a model that other institutions may adapt when integrating novel scientific content into the curriculum. Implementing a peer-informed approach to resource development (eg, the student-written primer) may also enhance perceived relevance and learning efficiency for medical students.

These implications extend primarily to curriculum design, instructional sequencing, and feasibility of implementation in undergraduate medical education. While the course addressed selected ethical and access-related issues within immunotherapy during discussions, the study did not evaluate growth in students’ understanding of social determinants of health or culturally competent care. Future research using targeted assessments could explore these domains more explicitly.

### Comparison With Literature

The integration of immunotherapy into undergraduate medical education remains limited, yet our findings mirror a growing body of evidence supporting earlier and more structured exposure to advanced cancer therapeutics. Several studies have reported that medical trainees feel underprepared to discuss immunotherapy, interpret mechanism-of-action data, or counsel patients about associated risks and benefits, a gap that our elective similarly sought to address [38]. Recent educational interventions have been developed during graduate medical education, including short courses, case-based modules, and interdisciplinary workshops.

Other research teams have emphasized the value of combining foundational basic science reviews with clinical case discussions to enhance long-term retention and clinical reasoning, an approach consistent with our spiral design and primer-supported structure [39,40]. These parallels support the relevance and effectiveness of our elective’s hybrid instructional format.

There is also a small but emerging literature on the importance of tailoring immunotherapy education to local contexts, including programs serving diverse or medically underserved communities. These studies underscore the need for cultural and demographic relevance when teaching advanced therapeutics, a theme that aligns with, but does not overextend beyond, the reflections shared by our students regarding practice readiness and communication skills [41-45].

Together, these converging findings suggest that structured, evidence-informed immunotherapy curricula can enhance medical students’ conceptual understanding and clinical preparedness. They also highlight the broader national momentum toward incorporating cutting-edge oncologic therapeutics earlier in medical training. Future multi-institutional studies will be essential to compare outcomes across diverse curricular models and to determine best practices for sustainable integration.

### Strengths and Limitations

A major strength of this elective was its alignment with up-to-date clinical and scientific standards in cancer immunotherapy. Due to the field evolving rapidly, faculty routinely reviewed and updated course content using evidence-based guidelines from the American Society of Clinical Oncology and the Society for Immunotherapy of Cancer. This process ensured that students engaged with current FDA-approved therapies, emerging research directions, and clinically relevant case examples, thereby reinforcing the scientific rigor and clinical applicability of the curriculum.

Another strength was the elective’s intentional design for a modular, hybrid structure, blending asynchronous learning with synchronous faculty-led discussion, allowing the course to adapt to varying institutional schedules and curricular models. In addition, its use of structured discussion prompts and a capstone innovation project created flexible instructional components that other medical schools could readily adopt or tailor to their own contexts.

The course also benefited from strong interdisciplinary collaboration. Faculty from immunology, oncology, biochemistry, clinical genetics, and internal medicine jointly developed and implemented the elective, ensuring cohesion across basic science and clinical content. Student cocreation, particularly in the development of the immunology primer, added a peer-informed dimension that enhanced clarity, relevance, and learner engagement.

Despite these strengths, several limitations warrant attention. First, participation in both the elective and the postcourse survey was voluntary, introducing potential self-selection and response bias that may limit the generalizability of the findings. Second, the retrospective nature of some perception-based survey items may have contributed to recall bias. Third, although the pre- and postcourse quizzes assessed knowledge gains, all data were analyzed at the aggregate level to maintain anonymity. The absence of individually paired responses limited our ability to conduct more detailed analyses of individual learning trajectories or matched-pair statistical testing.

The elective also faced implementation challenges, including variable student schedules, faculty workload constraints, and occasional technological barriers inherent to online instruction. Students on clinical rotations sometimes experienced difficulties synchronizing schedules with synchronous components. While some students pursued optional shadowing opportunities with oncologists, the elective did not include a formal clinical rotation, representing a potential area for future enhancement.

To address these challenges, we incorporated contingency measures such as recorded sessions, flexible deadlines, and alternative participation formats for discussion activities. Regular faculty meetings and scheduled check-ins with students allowed the instructional team to respond to emerging needs in real time. Attrition was minimal, as all enrolled students completed the elective; however, not all students completed the postcourse survey.

### Lessons Learned

Implementing this online cancer immunotherapy elective provided valuable insights into curriculum development, learner engagement, and program scalability. Early and meaningful collaboration between basic scientists, clinicians, and students was essential to ensure content relevance and clarity. Notably, the cocreation of the immunotherapy primer by medical students offered a peer-informed perspective that enhanced both accessibility and professional development for the authors.

Flexibility in course delivery, through asynchronous modules supplemented by live sessions, proved crucial for accommodating students’ clinical schedules and different learning styles. However, we learned that clear alignment between asynchronous and synchronous content was essential to avoid redundancy and promote continuity. Later course iterations were adjusted to better integrate these components.

Embedding structured opportunities for innovation, such as the final oral presentation task, fostered critical thinking and interdisciplinary application. Students were encouraged to propose novel therapeutic strategies, and faculty feedback emphasized this capstone experience as one of the most valuable elements of the elective.

Continuous improvement based on student feedback was also key. The anonymous survey asked students to identify strengths and areas for improvement, which led to enhancements in discussion prompts, session timing, and technical delivery. These efforts reinforced a culture of responsiveness and reflective teaching and demonstrated the importance of iterative design in building a sustainable, scalable intervention.

### Future Directions

Future research should explore how this elective contributes to long-term competence in cancer immunotherapy, especially during postgraduate training. While short-term outcomes demonstrated knowledge gain and student satisfaction, it remains critical to assess whether these improvements translate to clinical proficiency, leadership in immunotherapy initiatives, and patient-centered decision-making.

A key next step is longitudinal tracking of alumni who completed the elective, evaluating how early exposure to cancer immunotherapy influences clinical performance, advocacy, and continued professional development. This may include follow-up surveys, interviews, or program director assessments to gauge real-world application of course content.

Alternatively, conducting a cross-sectional cohort study comparing students who completed the elective with those who did not would offer insight into differences in long-term knowledge retention, confidence in discussing immunotherapy with patients, and likelihood of pursuing related research or clinical specialties.

To improve generalizability, future iterations of the elective should be piloted across additional medical schools with varying curricular structures and student demographics. Multi-institutional collaborations could help evaluate the adaptability, feasibility, and sustainability of this instructional model and identify institution-specific facilitators and barriers. Incorporating paired pre-post knowledge assessments or longitudinal tracking of clinical application skills may also strengthen future evaluation designs.

Expanding the elective to include interprofessional learners, such as pharmacy, nursing, and physician assistant students, could enhance team-based education and cancer care delivery. Finally, multisite implementation at diverse institutions will be critical for testing the generalizability and adaptability of the curriculum, and future iterations must remain agile to incorporate emerging therapies and updated guidelines in this rapidly evolving field.

## Supplementary material

10.2196/71628 Multimedia Appendix 1 Pre- and postelective quiz instrument. This quiz assessed medical students’ baseline and postcourse knowledge of cancer immunotherapy, including mechanisms of action, clinical applications, and immune-related adverse events. The same set of questions was administered before and after the elective to evaluate knowledge gains and support course effectiveness analysis.

10.2196/71628 Multimedia Appendix 2 Immunology primer overview. This primer provided foundational content on the immune system, including innate and adaptive immunity, key immune cells, cytokine signaling, and tumor immunology concepts. It was designed to ensure that students entered the elective with a baseline understanding necessary to engage in advanced cancer immunotherapy discussions.

10.2196/71628 Multimedia Appendix 3 Comprehensive impact of the immunotherapy course on medical students’ knowledge and critical thinking. (A) Students strongly agreed that the course was instrumental in learning current immunotherapy techniques, providing up-to-date content and practical applications that prepared them well for real-world settings. (B) The new immunotherapy course significantly enhanced students’ critical thinking in developing novel cancer treatments, with 19 out of 20 students affirming its impact. (C) Feedback from 20 medical students unanimously confirming that the content covered in the cancer immunotherapy course significantly filled gaps and deficiencies in their knowledge of immunotherapy. Each student reported agreement with the comprehensive nature of the course material, highlighting its effectiveness in addressing both fundamental concepts and advanced applications in the field of cancer treatment.

## Abbreviations

CAR: chimeric antigen receptor
CTLA-4: cytotoxic T-lymphocyte antigen 4
FDA: Food and Drug Administration
IRB: institutional review board
PD-1: programmed death-1
PD-L1: programmed death ligand-1
RE-AIM: Reach, Effectiveness, Adoption, Implementation, and Maintenance
TTUHSC: Texas Tech University Health Sciences Center
