# Supplementary material for: Implementation and Evaluation of a Cancer Immunotherapy Elective for Medical Students: Mixed Methods Descriptive Study
Source: JMIR Med Educ. 2026 Jan 21;12:e71628. doi: 10.2196/71628 (PMC12822871; doi:10.2196/71628)
Supplement: Multimedia Appendix 1 [file mededu-v12-e71628-s001.pdf]

Immunology Module Pre and Post-Assessment Questions

1. T/F: Tumor suppressor genes are genes whose products are indirectly involved in turning off the cell cycle.

2. Which Tumor Suppressor Gene is known as the Guardian of the Genome?
   a. *Rb*
   b. *P53*
   c. *INK4*
   d. *PTEN*
   e. *BRCA1*

3. Which of the following best explains the "two-hit hypothesis"?
   a. One of the tumor suppressor gene copies must be defective for cancerous proliferation to occur.
   b. It is required that both copies of a tumor suppressor gene must be defective for pro-cancerous effects to occur.
   c. A single copy of a tumor suppressor gene is insufficient to maintain normal cellular processes.
   d. Describes why not all women with a BRCA1/2 mutation get breast cancer.
   e. Both B and D

4. Fill in the Blank: The ________ gene is a "signal hub" that promotes downstream signaling in multiple pro-growth pathways.
   a. PI3K
   b. RAF
   c. Ral GEF
   d. K-RAS

5. T/F: Negative Selection takes place in the medulla of the Thymus.

6. In the cortex of the Thymus, positive selection occurs through T-cell receptor sampling of peptides. If the T-cell receptor is defective and binds too strongly or weakly to the presented self-antigen, the T-cell will undergo which cellular process?
   a. Apoptosis
   b. Necrosis
   c. Autophagy
   d. Autolysis

7. Which of the following do active CD-8 T-cells release?
   a. Perforin
   b. Granzymes
   c. C3b
   d. Both A and B

e. Phagolysosomes

8. Which surface cell marker is increased in frequency during tumorigenesis?
    a. PD-L1
    b. CD28
    c. CD3
    d. CD25
    e. CYP11A1

9. A significant component in cancer vaccines includes a method for producing memory T cells and memory B cells that are sensitive to specific antigens and undergo massive proliferation to fight cancer cells. This more robust immune response is due to which of the following?
    a. Helper T Cells
    b. Adjuvant Compounds
    c. Biomarker Enhancing Compounds
    d. Anti-PD-1

10. When Cancer cells downregulate MHC class 1 complexes, which of the following does the immune system rely on as backup? Hint: This cell causes granzymes-mediated destruction of the tumor cells.
    a. Cytotoxic T Cells
    b. Macrophages
    c. Natural Killer Cells
    d. Neutrophils
    e. Memory B Cells

11. Select all that apply: Which of the following are essential cytokines in the Tumor Microenvironment that promote a *pro-inflammatory state*?
    a. IL-1
    b. IL-6
    c. TNF-α
    d. IL-10
    e. IL-12
    f. PDGF
    g. IL-4
    h. TGF-β

12. Select all that apply: Which of the following are essential cytokines in the Tumor Microenvironment that promote *immunosuppression*?
    a. IL-1
    b. IL-6
    c. TNF-α
    d. IL-10

e. IL-12
f. PDGF
g. IL-4
h. TGF-β

13. Which compounds allow tumor cells to promote angiogenesis, thus developing a blood supply for growing tumors?
    a. PDGF
    b. VEGF
    c. IFN-γ
    d. IL-2

14. Cytokine therapy has become more advanced as researchers further understand tumor microenvironments. This therapy has been a staple in the management of many cancers. Which cytokines have been FDA-approved to enhance the proliferation of immune cells?
    a. IL-2
    b. IL-4
    c. TGF-β
    d. IL-10

15. T/F: CAR-T therapy extracts a sample of a patient's T-cells and inserts genes in-vitro that code for a chimeric antigen receptor, thus designed to bind specific proteins on cancer cells.
