# Supplementary material for: Implementation and Evaluation of a Cancer Immunotherapy Elective for Medical Students: Mixed Methods Descriptive Study
Source: JMIR Med Educ. 2026 Jan 21;12:e71628. doi: 10.2196/71628 (PMC12822871; doi:10.2196/71628)
Supplement: Multimedia Appendix 2 [file mededu-v12-e71628-s002.pdf]

Copyright TTUHSC 2023

Immunology Primer: The Inter-Play Between Cancer and the Immune System

Created by: Rivers Hock MS3

Images created by: Jessica Chacon, Ph.D.

A cancer cell is the product of our own "self-cells" that have undergone extensive mutations and are experiencing unregulated cellular divisions. This can occur through a mutation within a **tumor suppressor gene** or a **protooncogene.**

Tumor suppressor genes are genes whose products are directly involved in shutting off the cell cycle. One of the most prominent examples of tumor suppressor gene is **p53** also known as the **"guardian of genome"**, p53 is involved in shutting off the cell cycle and promoting DNA repair or apoptosis to cells that exhibit signs of mutation or DNA damage. This prevents mutated cells from re-entering the cell cycle and accumulating further mutations and eventually forming a cancer cell. Tumor suppressor mutations are known to follow "**the two-hit hypothesis,"** this refers to the concept that both copies of a tumor suppressor gene must be defective for pro-cancerous effects to occur. This is due to the fact, that a single copy of a tumor suppressor gene can often be sufficient in maintaining normal cellular processes.

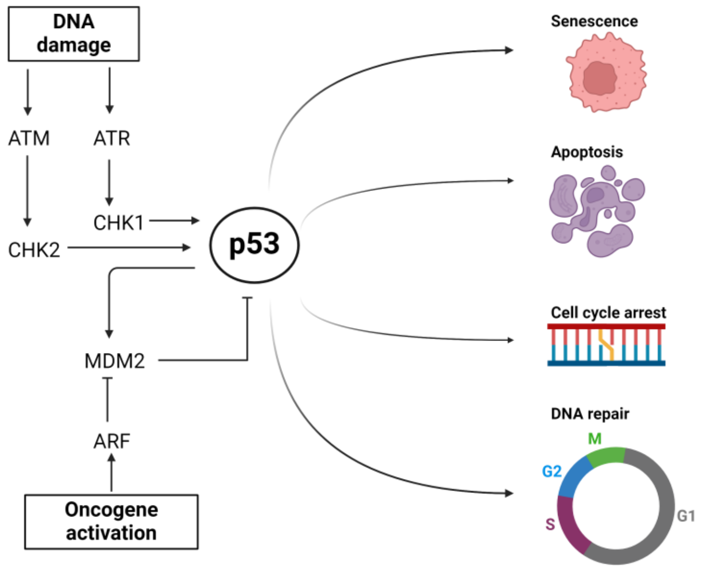

Image created by Biorender.

Protooncogenes are genes that promote the entry into cell cycle. One of the most prominent examples of a protooncogene is the **K-RAS** gene. The K-RAS gene is a "signal hub" that promotes downstream signaling in multiple pro-growth pathways. The RAS protein is turned "on" by being maintained in its **GTP state** and can be turned off by removal of a phosphate group to the **GDP state**. Several mutations have been identified that cause the RAS protein to favor the GTP state. This triggers a downstream signaling cascade by the RAS protein that promotes cellular growth, survival, and proliferation. These are all conditions that favor cancer cell formation. As opposed to tumor suppressor genes, it is common for a single copy of a mutation in a protooncogene mutation to promote pro-cancerous effects.

Copyright TTUHSC 2023

How Does the Immune System Recognize Cancer Cells?

A widely known concept regarding the immune system is the concept of **“friend versus foe”**. **The thymus** is an organ within the body that promotes the proper development of **T cells**. In the **cortex** of the thymus **positive selection** occurs in which the T-cell receptor is tested against a sampling peptide that is presented on a thymic epithelial cell. If the T-cell receptor is defective and is unable to bind the presented antigen, the T-cell will undergo **apoptosis**. This prevents the defective T-cell from persisting and going on to cause **autoimmune** related complications.

Within the **medulla** of the thymus, negative selecction takes place. In negative selection, the **AIRE gene** is responsible for producing self antigens which are then loaded onto **MHC complexes** on **antigen presenting cells**. T-cells are then exposed to to these antigens. If the T-cells bind strongly it indicates that the T-cells are reactive against self peptides and the T cells undergo apoptosis. If the T-cells fail to bind they persist, leave the thymus, and enter general circulation to mature.

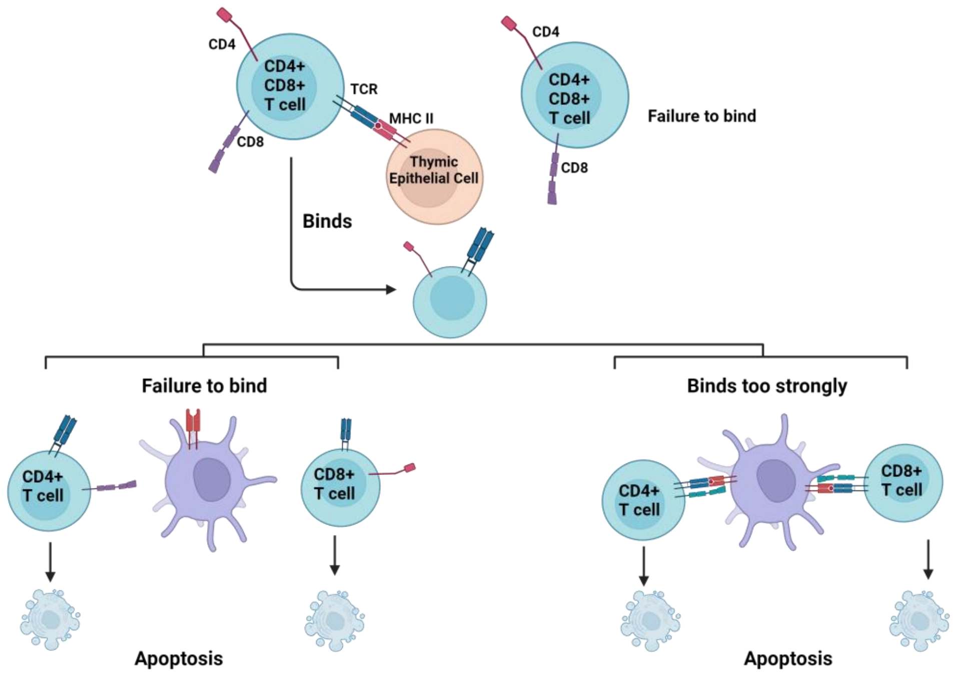

Image created by Biorender

As explained, T cell are **“educated”** in the thymus to be non-reactive to self-proteins. We have also explained that cancer cells are self-cells that have merely undergone mutation and are experiencing unregulated cellular divisons. This begs a very important question, **“How does the immune system recognize cancer cells if they are self-cells with self-antigens?”**

This connects back to a central component of cancer cells. They have accumulated significant mutations. These mutations have the capacity to drastically impact cellular functions and processes. One of these processes is often **protein processing and production**. These irregularities in protein formation have the

Copyright TTUHSC 2023

potential to create two unique antigens subtypes that allow T-cells to recognize tumor cells as abnormal and subsquently generate an inflammatory reaction. **Tumor-specific antigens** are proteins that by means of mutation and cancer formation are unique to only tumor cells and are not found on normal “self cells”. **Tumor associated antigens** are proteins that can be found on normal “self cells” but are formed by

1.) the reactivation of **embryonic genes** in fully differentiated cells or 2.) a particular protein is overexpressed and alters the **density of MHC expression** compared to baseline.

T-Cell Recognition of MHC Complexes

MHC class I complexes are found all nucleated cells and are loaded with antigen proteins found within the cell. The MHC class I complex interacts with **T-cell receptors** on **CD-8 T cells**. The T-cell then undergoes downstream signaling that promotes the release of **perforin and granzymes.** These compounds perforate the cell membrane and degrades proteins causing **apoptosis**. This process occurs when a T-cell recognizes a **tumor-associated antigen** or **a tumor specific antigen** loaded onto an MHC class 1 complex.

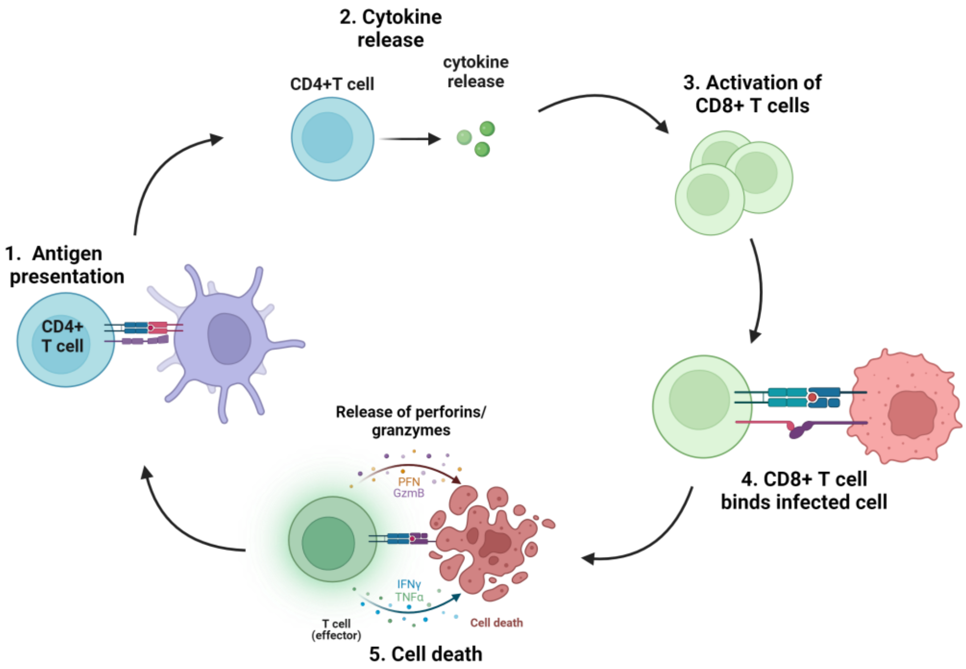

Image created using Biorender.

Cancer Cell Adaptations to Avoid the Immune System

Given that cancer cells have **unstable genomes** and rapidly divide, these cells have an increased capacity to gain **mutations** or **adaptations** in cellular biology that may help the cancer cells survive and persist. As described above, a major barrier that cancer cells have towards survival is the immune response. Therefore, many cancer cells undergo alterations that allow them to avoid the immune system. One example of this is that cancer cells frequently **downregulate MHC class I complex** on the cell surface. As explained, the MHC class I complex is loaded with self proteins and allows **cytotoxic CD8 T cells** to sense

Copyright TTUHSC 2023

if there are any **tumor specific antigens** or **tumor associated antigens** being produced by the cell. If the MHC class I complex is downregulated the **CD8 T cell** has no way of sensing these antigens. Therefore, **perforin and granzyme** are not released onto the tumor cell and the cancer cell has avoided **a CD8 T cell mediated immune response.**

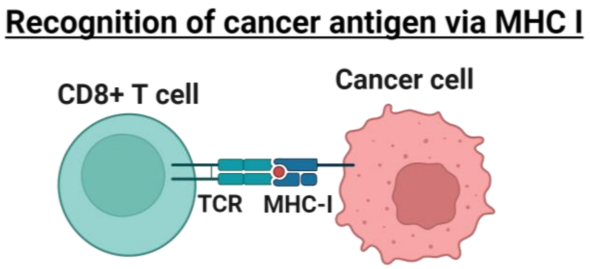

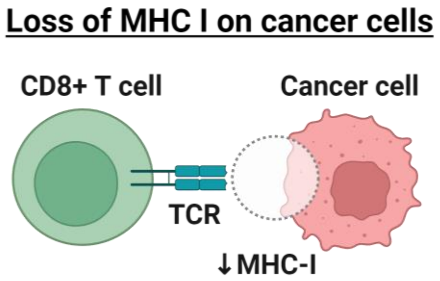

Image created using Biorender.

Tumor cells also are known to increase the density of complexes on their cell surface in order to avoid the immune system. One complex that is frequently increased on the surface of tumor cells is **PD-L1** or **the programmed cell death ligand 1**. This ligand is found on a wide array of immune cells are normal cell types but is massively expressed on the surface of numerous cancer cells. **PD-L1** is known to bind **PD-1** or **programmed death receptor 1** which is widely present on **CD8 T cells**. This creates an inhibitory signal within **CD8 T cells**. This often results in anergy and overall **T-cell inactivation**

PD-1 Antibodies: Checkpoint Inhibitors

To overcome tumor cells with a high density of **PDL-1** on their cell surface, researchers have engineered **antibodies** that bind to **PD-1 or programmed death receptors** on the surface of **T cells.** These antibodies (the most known being **embrolizumab, nivolumab, and durvalumab**) blocks the binding site between **PDL-1 and PD-1**. This prevents **T cells** from being inactivated and undergoing **anergy.** Therefore **T-cells** remain more active in the **tumor microenvironment** and promote destruction of cancer cells.

Copyright TTUHSC 2023

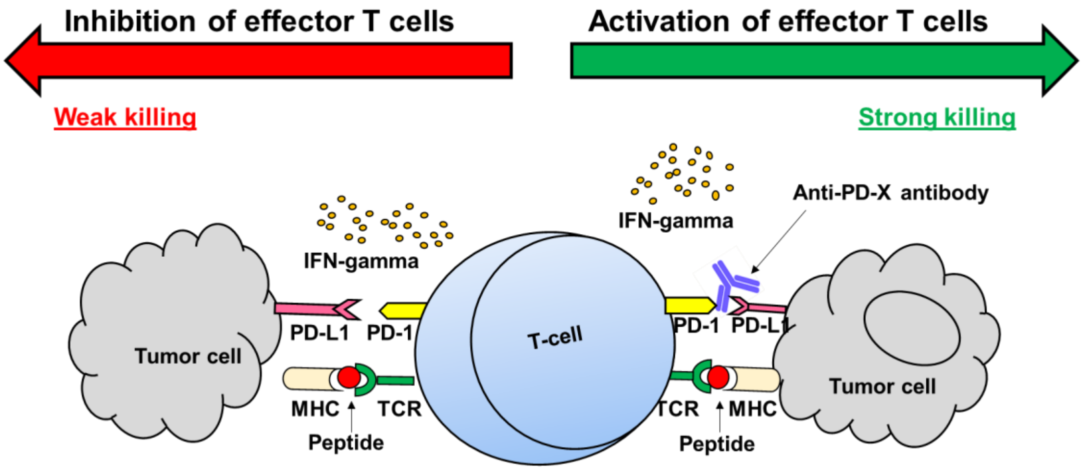

Utilizing Tumor Antigens in Vaccines

Given that **tumor associated antigens** and **tumor specific antigens** can be recognized by the immune system. Researchers have begun to work on developing **cancer vaccines** that include these antigens alongside **adjuvant compounds**. This is meant to produce **memory T cells** and **memory B cells** that are sensitive to these antigens and undergo massive proliferation to fight cancer cells.

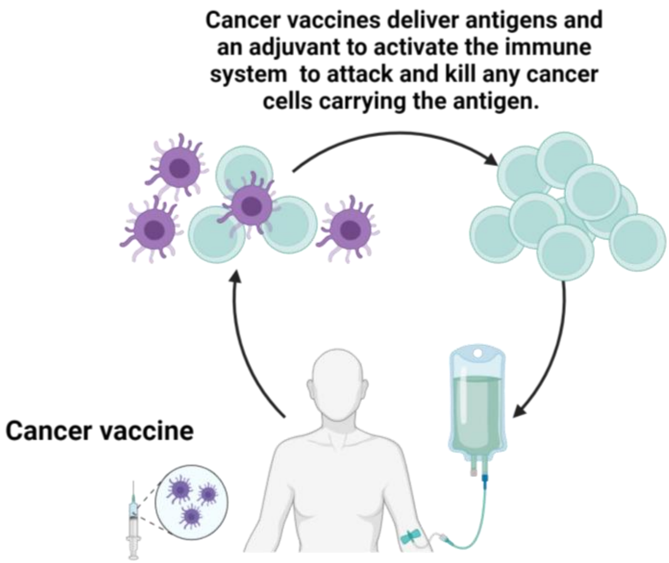

Image created using Biorender.

NK Cells: Cancer Killers

If cancer cells often drastically downregulate **MHC class I complexes** dramatically T cells become less effective at initiating an immune response. At this point, the immune system begins to heavily rely on a different cell type called **NK cells** or **natural killer cells**. These cells are programmed to release granzymes on any nucleated cell detected to l ack an **MHC class I complex**. This occurs through a

Copyright TTUHSC 2023

twostep system where an **activating ligand** on a nucleated cell binds to an **activating receptor** on **NK cells**. This generates an activating pro-inflammatory signal within the **NK cell.** This pro-inflammatory signal can only be "turned off" by **the inhibitory receptor** on **NK cells** binding to the **MHC class I complex.** Therefore, tumor cells that lack an **MHC class 1 complex** on the cell surface do not have the capacity to turn off the **pro-inflammatory signal** initiated by the **activating receptor** on **NK cells** causing **granzyme** mediated destruction of the tumor cells.

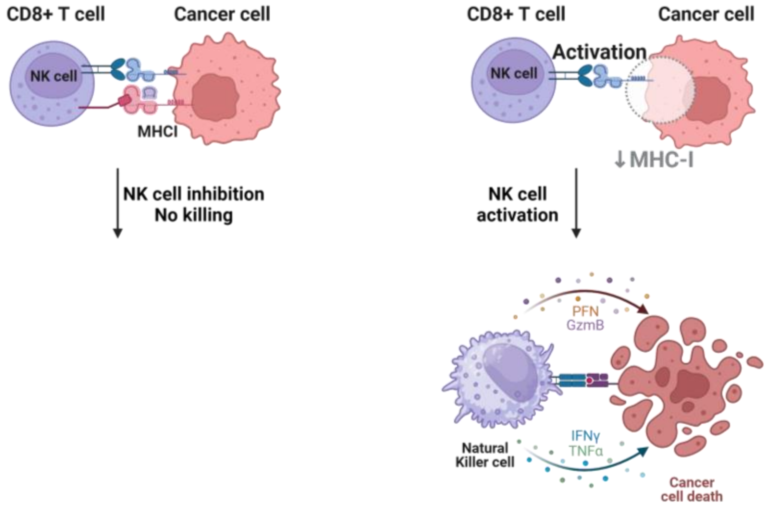

Image created using Biorender.

<u>The Tumor Microenvironment</u>

Cancer cells are suspended in a small local environment that contain numerous **immune cells**, **cytokines**, **chemokines,** and **other factors** that influence the strength of the local immune response. Cytokines such as **IL-1, IL-6,** and **TNF alpha** are known to promote a **pro-inflammatory state.** On the other hand, cytokines such as **IL-10** and **TGF-beta** are known to promote **immunosuppression.** Clearly, tumor cells favor an **immunosuppressive environment** as this prevents immune cells from initiating inflammatory events that promote tumor cell destruction. Tumor cells are capable of directly manipulating the **tumor microenvironment** to favor an immunosuppressive environment. One method of doing this is by hijacking the differentiation of **macrophages** within **the tumor microenvironment** to favor the **M2 phenotype. M2 macrophages** favor the release of **IL-10** and **TGF-beta**. By favoring the **M2 phenotype** less **M1 macrophages** are produced which release inflammatory cytokines such as **IL-6 a**nd **TNF-alpha.**

Copyright TTUHSC 2023

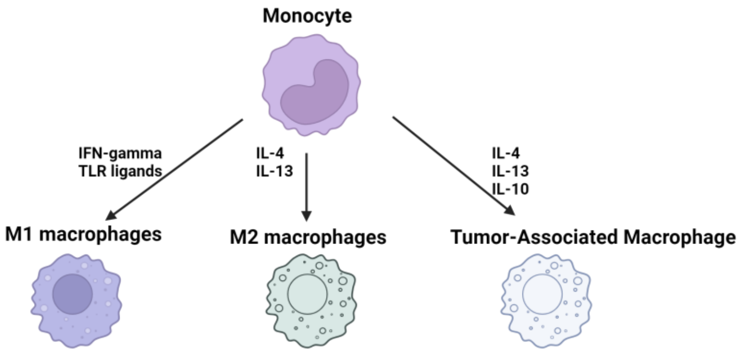

	M1 macrophages	M2 macrophages	Tumor-Associated Macrophage
**Expressed Markers**	MHC-II, CD68 CD80, CD86, iNOS	CD163, CD206, Arg1 CD200R, MGL-1 MGL-2	CD163, CD206, Arg1 FcR
**Secreted factors**	IL-6, IL-12, IL-23, TNF-alpha	IL-10, TGF-beta	IL-10, TGF-beta, CCL2, CCL5
**Function**	Pro-inflammatory Cytotoxic Anti-tumorigenic	Immunosuppressive Pro-tumorigenic Anti-inflammatory	Immunosuppressive Pro-tumorigenic

Image created using Biorender.

Tumor cells are also known to directly release compounds that have **immunosuppressive effects.** An example includes **VEGF** or **vascular endothelial growth factor**. This compound allows tumor cells to promote **angiogenesis** in order to develop a **blood supply** for a growing tumor. **VEGF** is also known to promote the proliferation of **immunosuppressive cells**.

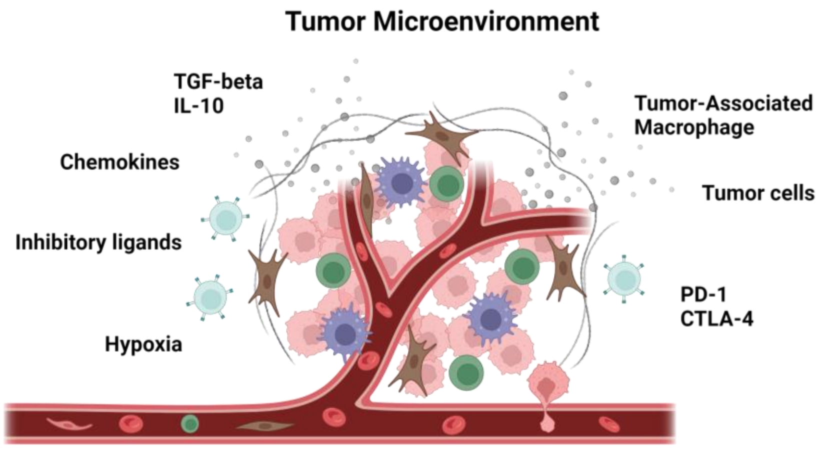

Image created using Biorender.

<u>**Cytokine Therapy: Overcoming the Tumor Microenvironment**</u>

Understanding how tumor cells manipulate the microenvironment allows you to understand one major **immunotherapy. Cytokine therapy** is a staple in the management of many cancers. For instance, direct

Copyright TTUHSC 2023

IL-2 administration is FDA approved for the treatment of **renal cell carcinoma and metastatic melanoma**. **IFN-alpha** has been approved in the treatment of **hairy cell leukemia, follicular non-Hodgkin lymphoma, melanoma, and AIDS related Kaposi sarcoma.** Administering these cytokines vamps up and bolsters the immune system to favor a **pro-inflammatory state**.

CAR-T Therapy: Cell Engineering

As technology and science have improved researchers have begun efforts to design "super soldier" Tcells specifically engineered to target and kill cancer cells. This technique is called **CAR-T therapy** and is initially started by extracting a sample of a patients own T-cells. These T-cells are then taken to a laboratory where a scientist inserts genes into the T-cells that codes for a **chimeric antigen receptor**. Chimeric antigen receptors are special receptors created in the laboratory that are designed to bind specific proteins on cancer cells.

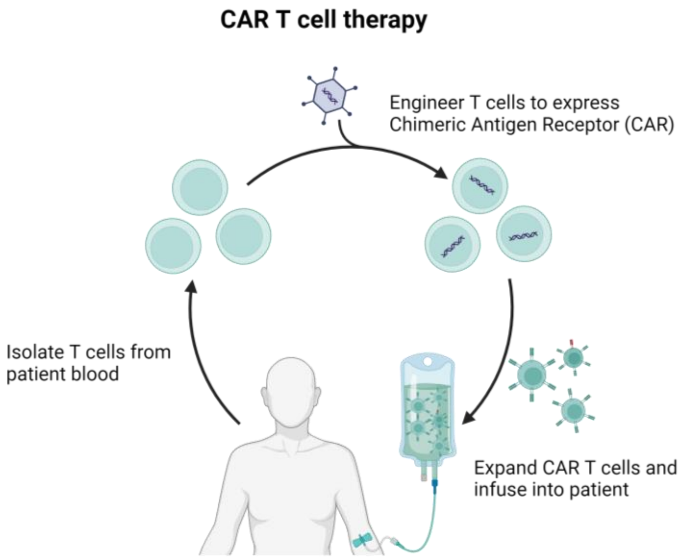

Image created using Biorender.
