# Supplementary figures and images for: Implementation and Evaluation of a Cancer Immunotherapy Elective for Medical Students: Mixed Methods Descriptive Study
Source: JMIR Med Educ. 2026 Jan 21;12:e71628. doi: 10.2196/71628 (PMC12822871; doi:10.2196/71628)

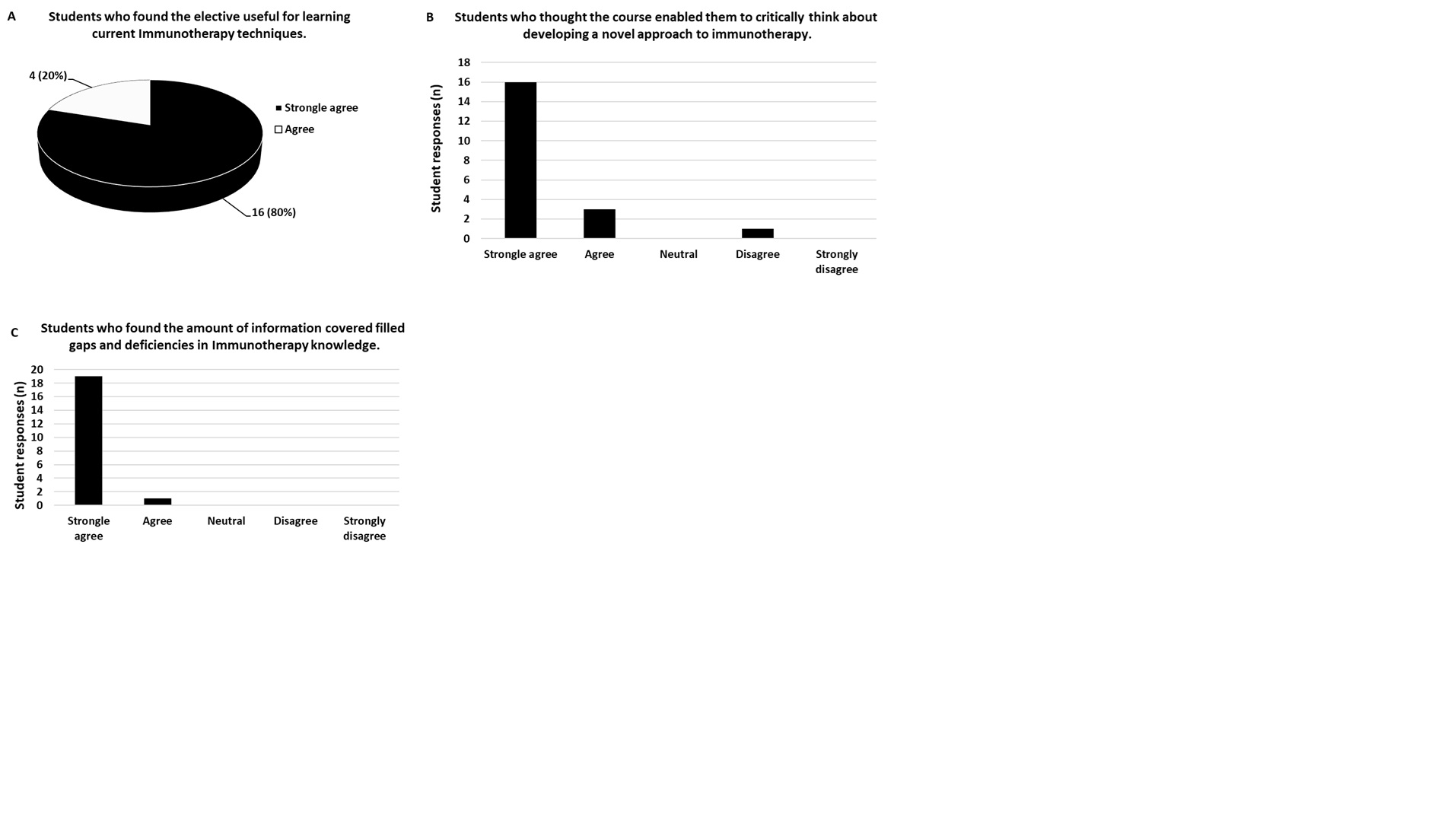

Supplement: Multimedia Appendix 3 [file mededu-v12-e71628-s003.jpg]
